# Is body mass index associated with irregular menstruation: a questionnaire study?

**DOI:** 10.1186/s12905-020-01085-4

**Published:** 2020-10-08

**Authors:** Yunhui Tang, Yan Chen, Hua Feng, Chen Zhu, Mancy Tong, Qi Chen

**Affiliations:** 1 Department of Family Planning, The Hospital of Obstetrics & Gynaecology, Fudan University, Shanghai, China; 2 Department of Obstetrics & Gynaecology, Tengzhou Central People’s Hospital, Tengzhou, Shandong Province China; 3 Department of Obstetrics, Gynaecology and Reproductive Sciences, Yale School of Medicine, New Haven, CT USA; 4 The Department of Obstetrics & Gynaecology, The University of Auckland, Auckland, New Zealand

**Keywords:** BMI, Irregular menstrual cycle, Menstrual blood loss, Chinese women

## Abstract

**Background:**

Irregular menstrual cycles including the length of cycles and menses, and heavy menstrual blood loss are linked to many gynaecological diseases. Obesity has been reported to be associated with irregular menstrual cycles. However, to date, most studies investigating this association are focused on adolescence or university students. Whether this association is also seen in adult women, especially women who had a history of birth has not been fully investigated.

**Methods:**

Questionnaire data were collected from 1012 women aged 17 to 53 years. Data on age, weight and height, gravida, the length of menstrual cycles and menses, and the number of pads used during menses were collected. Factors associated with menstrual cycle according to BMI categories were analysed.

**Results:**

There were no differences in the length of menstrual cycles and menses in women of different body mass index (BMI) groups. However, there was a significant difference in menstrual blood loss in women of different BMI categories. The odds ratio of having heavy menstrual blood loss in obese women was 2.28 (95% CL: 1.244, 4.193), compared to women with normal weight, while there was no difference in the odds ratio of having heavy menstrual blood loss in overweight, compared to normal weight, women. In contrast, the odds ratio of having heavy menstrual blood loss in underweight women was 0.4034 (95% CL: 0.224, 0.725), compared to women with normal weight.

**Conclusion:**

Although BMI was not correlated with the length of menstrual cycle and menses, BMI is positively associated with menstrual blood loss. Our data suggest that BMI influences menstrual blood loss in women of reproductive age and weight control is important in women’s reproductive years.

## Background

The menstrual cycle is a repetitive and regular natural change that occurs during the reproductive ages in women. This involves changes in endometrial structure, function and reproductive hormone production. A number of factors that often play a role in the regularity and blood loss of a woman’s menstrual cycle include female sex hormone changes, genetics, serious medical conditions, body mass index (BMI), lifestyle and stress.

Obesity is becoming a growing public health issue and is one of the leading causes of morbidity and mortality in many diseases. Obesity also increases the risk for developing gynaecological diseases including infertility and menstrual dysfunction [1], although a variety of sex hormones also play an important role in these disorders. These gynaecological diseases include a wide range of abnormalities including the length of menstrual cycle, length of menses, irregular menstrual cycle and menstrual blood loss. It was reported that obese women had more irregular and longer menstrual cycle [2–8]. A questionnaire study showed a rate of approximately 3% heavy menstrual bleeding in women aged 30 to 49 years [9]. Although there was no BMI data in that study, heavy menstrual blood loss was often seen in overweight women [2, 10]. However, the population studied included in most of these studies were adolescence or university students, which may not be able to be extrapolated to adult women. This is because age is associated with the length of menstrual cycle [11].

There is growing evidences also suggesting that menstrual cycle dysfunction in women is linked to breast cancer, endometrial cancer, cardiovascular disease and neurologic disorders [12–14]. The risk of developing endometrial hyperplasia is significantly increased in overweight women. This finding resulted in The National Institute for Health and Care Excellence (NICE) recommending a lower threshold for endometrial biopsies, when overweight women have persistent intermenstrual bleeding (NICE Clinical guideline 44: heavy menstrual bleeding, 2007). Our recent study also reported that BMI is negatively associated with the stage of endometrial cancer, which may be due to early endometrial biopsy in overweight women with abnormal bleeding resulting from the NICE guideline [15].

Although a few studies have reported that obesity is associated with irregular or longer menstrual cycles or amenorrhea in women at a young age, studies about the association between BMI and heavy menstrual blood loss are still not well documented in women of a reproductive age. A cross-section study reported that women with higher BMI using Mirena intrauterine system had a longer time interval to be diagnosed with amenorrhoea and then consequently suggested that women with higher BMI were likely to have heavier menstrual blood loss [16]. In addition, Asian woman have a relatively lower BMI in comparison to Caucasians. Therefore, in this study we investigated the association between BMI and the length of menstrual cycle, menses and menstrual blood loss in Chinese women aged 17 to 53 years.

## Methods

This questionnaire study was performed in The Hospital of Obstetrics & Gynaecology of Fudan University, Shanghai, China from August 2018 to December 2018 and Tengzhou Central People’s Hospital of Shandong Province, China from October 2018 to December 2018. This study was approved by the ethics boards of The Hospital of Obstetrics & Gynaecology of Fudan University and Tengzhou Central People’s Hospital.

### Patient and public involvement

1012 women were involved in participating in this questionnaire study.

### Study participants

A total of 1100 women who came to our hospitals for routine gynaecological examination were randomly selected and asked to voluntarily complete a questionnaire. The questionnaire developed for this study is provided as Additional file 1 (supplementary file). Women with any chronic gynaecological disorders that could potentially affect the menstrual cycle and women with any endocrinological disorders including diabetes were excluded. In addition, women who had a pregnancy within 12 months were also excluded. All these women voluntarily completed the questionnaire. The questionnaire included age, weight and height, parity and gravida, the average length of menstrual cycles, the length of menses and the number of pads used during menses. With 92% response rate, 1012 questionnaires were returned and analysed in this study.

To measure menstrual blood loss, a pictorial blood loss assessment chart (PBAC) was described in 1990 [17]. However, a recent validation study reported that there was a poor correlation between observed PBAC score and menstrual blood loss [18]. To date there is no well described standard method to measure menstrual blood loss. Therefore, in our current study, we divided the amount of menstrual blood loss into three groups, light (less than 15 pads used during menses), medium (15 to 20 pads used during menses) and heavy (more than 21 pads used during menses) based on our gynaecologist’s clinical experience.

BMI was calculated and classified according to the ethnicity-specific WHO classification [19] for Asian women: underweight (under 18.4 kg/m^2^), normal weight (18.5–22.99 kg/m^2^), overweight (23–27.49 kg/m^2^), and obese (over 27.50 kg/m^2^).

### Statistical analysis

Statistical difference in the amount of menstrual blood loss according to BMI was assessed with Chi-square test using the Prism software package. The analysis in the rate of women with heavy menstrual blood loss was assessed by odds ratio and 95% confidence limits (CL) using OpenEpi software. The statistical difference in pads used during menses between women with normal weight and underweight women, or overweight women or obsess women was assessed with a Mann-Whitney U-test using the Prism software package. *P*-values of < 0.05 were considered significant.

## Results

### General characteristics of the study population

The general characteristics of study participants are summarised in Table 1. The median age of women was 30 (range 17–53) years old. There was no difference in age among groups according to BMI categories (Table 1, *p* > 0.05). Of 1012 women, 749 (74%) women had at least one previous pregnancy, and 299 (30%) women were overweight or obese.

**Table 1:** General parameters of study cohort

Age (years, median/range)	30 (17–53)
Underweight	27 (18–45)
Normal weight	30 (17–51)
Overweight	33 (17–53)
Obese	31 (19–51)
Gravida (number, %)	
0	263 (26%)
> 1	749 (74%)
BMI (kg/m^2^)	
Underweight (number, %)	121 (12%)
Normal weight (number, %)	592 (58%)
Overweight (number, %)	252 (25%)
Obese (number, %))	47 (5%)

### BMI was associated with the amount of menstrual blood loss

There was no difference in the average length of menstrual cycle and menses among groups according to BMI categories (Table 2). However, the amount of menstrual blood loss was significantly different among the four groups according to BMI categories (Table 2, *p* = 0.001, Chi-square test). We then selected women with normal weight as a referent group to compare with other groups (Table 3). The odds ratio of women with heavy menstrual blood loss in obese women was 2.28 (95% CL: 1.244, 4.193), compared to that in women with normal weight (*p* = 0.004). The odds ratio of women with heavy menstrual blood loss in overweight women was 1.26 (95% CL: 0.903, 1.749), compared to that in women with normal weight (*p* = 0.088). In contrast, the odds ratio of women with heavy menstrual blood loss in women with normal weight was 2.479 (95% CL: 1.378, 4.462), compared to that in underweight women (*p* = 0.0005).

**Table 2:** Analysis of menstrual blood loss according to BMI categories

BMI categories	Length of cycle (Days, median/range)	Length of menses (Days, median/range)	Menstrual blood loss			
			Light (number, %)	Medium (number, %)	Heavy (number, %)	Chi-square test^a^
Underweight (*n* = 121)	30 (23–60)	6 (3–8)	61 (50%)	46 (38%)	14 (12%)	P = 0.001
Normal weight (*n* = 592)	30 (23–60)	5.5 (2–8)	241 (41%)	206 (35%)	145 (24%)	
Overweight (*n* = 252)	30 (23–60)	5.5 (2–10)	92 (36%)	87 (34%)	73 (29%)	
Obese (*n* = 47)	29 (20–35)	5 (3–7)	15 (32%)	12 (26%)	20 (42%)	

^a^Chi-square test was performed for analysis of the amount of menstrual blood loss and BMI

**Table 3:** Analysis of women with heavy menstrual blood loss compared to women without heavy menstrual blood loss according to BMI categories

BMI categories	Heavy (number, %)	Not heavy (number, %)	Odds Ratio	95% CL	*P* value
Normal weight (reference) (n = 592)	145 (24%)	447 (76%)	–	–	**–**
Obese (n = 47)	20 (42%)	27 (58%)	2.28	1.244, 4.193	**0.004**
Overweight (n = 252)	73 (29%)	179 (71%)	1.26	0.903,1.749	0.088
Underweight (n = 121)	14 (12%)	107 (88%)	0.4034	0.224,0.725	**0.0005**

*CL* confidence limits

Because there was no difference in the length of menses, we then analysed the number of pads used during menses according BMI (Table 4). The average pads used per day during menses in overweight or obese women were significantly more than that in women with normal weight (*p* = 0.003, or *p* = 0.0012, respectively), while women with underweight used significantly fewer pads per day compared to women with normal weight (*p* = 0.0001).

**Table 4:** Analysis of pads used during menses according to BMI categories

BMI categories	Pads/day (number, mean ± SD)	P value (Mann-Whitney test)
Normal weight (n = 592) (reference)	2.75 ± 0.86	–
Obese (n = 47)	3.21 ± 1.00	P = 0.0012
Overweight (n = 252)	2.93 ± 0.90	P = 0.003
Underweight (n = 121)	2.43 ± 0.77	P = 0.0001

## Discussion

In this questionnaire study with a relatively large sample size, we found that BMI is not correlated with the length of menstrual cycles and menses, but BMI is positively associated with menstrual blood loss in adult women.

To date, most studies investigating the association between BMI and abnormalities of the menstrual cycle focused on a younger women population, aged between 18 to 25 years and there was no information on history of childbirth [2–8]. Age is associated with the length of menstrual cycle and women over 35 years had shorter menstrual cycles [11]. In addition, the association between BMI and menstrual blood loss has not been fully investigated, although a high BMI increases the risk of ovulatory dysfunction and subsequently may cause heavy menstrual blood loss [2]. In our current study, we investigated the association between BMI and menstrual cycle characteristics in women with a wide range of ages (aged 17 to 53 years), using ethnicity-specific WHO classification of BMI. This could represent such associations in women of a reproductive age.

Although a variety of sex hormones play an important role in affecting menstrual cycles, studies have suggested that having a high BMI may cause an absence of menstruation, irregular menstruation, heavy or long menstruation and painful menstruation [2–6, 8]. In our current study, we found that 25% of Chinese women had heavy menstrual blood loss which is consistent with a previous study that reported 28% of adolescents had heavy menstrual blood loss. While another study with self-reported data only showed 3% of women with heavy menstrual blood loss [9], the difference in age between our study and that study could be one of the contributing reasons. In our current study, we investigated women aged 17 to 53 years, while in that study, they only investigated women aged 30 to 49 years. A previous study has reported that women over 35 years had shorter menstrual cycle [11].

To date, studies about the association between BMI and menstrual blood loss are very limited since most studies investigated the association between BMI and irregular menstrual cycle lengths [2–8]. A previous study reported a higher incidence of heavy menstrual blood loss and longer menstrual cycle and irregular menstrual cycle in overweight women [2]. However, in that study they did not individually investigate such associations. In our current study, we found that the odds ratio of having heavy menstrual blood loss in obese women was 2.28 (95% CL: 1.244, 4.193), compared to women with normal weight. The odds ratio of having heavy menstrual blood loss in overweight women was 1.26 (95% CL: 0.903, 1.749), compared to women with normal weight, but this odds ratio did not reach to statistical significance (*p* = 0.08). In addition, we also found that the number of pads used during menses was significantly more in obese women, compared to women with normal weight. Because there was no difference in the length of menstrual cycle and menses in subgroups according to BMI categories in our current study, taken together our data may suggest that obese women are more likely to experience heavy menstrual blood loss, which supports a previous hypothesis [16].

The underlying mechanism of our findings is unclear, but it may be explained by obesity being associated with excessive levels of estrogen [20, 21], which is the main sex hormone affecting menstrual cycles including menstrual blood loss. Women with very low body fat had significantly lower levels of estrogen [22]. It has been suggested that weight loss can restores regular menstrual function by decreasing the aromatisation of androgens to estrogen in adipose tissue. As obese women commonly have a degree of insulin resistance, a study has found that metformin, a medicine to treat insulin resistance, can restore normal menses in women with heavy menstrual blood loss [23]. In our current study, we also found that the number of women with heavy menstrual blood loss was significantly lower in underweight women than in women with normal weight (The odds ratio of having heavy menstrual blood loss in underweight women was 0.4034 (95% CL: 0.224, 0.725), compared to women with normal weight). Taken together, our data further suggests that adipose tissue may be associated the menstrual blood loss through estrogen production. Our current finding also could support our recent published study which showed a negative association between BMI and the stage of endometrial cancer [15]. Abnormal vaginal bleeding is one of the main clinical symptoms of endometrial cancer in both premenopausal and postmenopausal women, and increased unopposed estrogen (which can be associated with obesity) is associated with abnormal vaginal bleeding.

In our present study we did not find any difference in both the length of menstrual cycles and the length of menses among groups according to BMI categories, although previous studies found such association [2–7]. Again, the difference could be explained by the age of women who participated in these studies. In our present study we included a wider range of ages in women who participated.

We acknowledge that there are some limitations in this study. Firstly, all data including weight and height were self-reported which may induce bias, in particular in recalling the number of pads used during menses. There is also a potential bias on the different type of pad with different absorbent capacity used. Second, we do not have data on smoking, physical activity and alcohol consumption, as smoking and alcohol consumption can cause hypoestrogenism [24] and shorten the menstrual cycle [11]. But the prevalence of women who are smoking or who drink is relatively low in China based on the Chinese traditional culture. In addition, data were obtained from two hospitals from different regions, and BMI and economic levels may vary in China. Therefore, our data may not be representative of China as a whole.

## Conclusion

In this study, we found that BMI is positively associated with menstrual blood loss and in contrast, BMI is not correlated with the length of menstrual cycles and menses in our study population. Since heavy menstrual blood loss can limit physical and social activities, our data suggests that weight control is important for improving a woman’s quality of life during her reproductive years.

## Supplementary information

**Additional file 1.** Questionnaire.

## Data Availability

The datasets used and/or analysed during the current study available from the corresponding author on reasonable request.

## Abbreviations

BMI: Body mass index
NICE: National Institute for Health and Care Excellence
PBAC: pictorial blood loss assessment chart
