# Supplementary material for: Is body mass index associated with irregular menstruation: a questionnaire study?
Source: BMC Womens Health. 2020 Oct 8;20:226. doi: 10.1186/s12905-020-01085-4 (PMC7545932; doi:10.1186/s12905-020-01085-4)
Supplement: Supplementary file 1 — Additional file 1. Questionnaire. [file 12905_2020_1085_MOESM1_ESM.doc]

**Questionnaire**

**Dear Madam**

We are undertaking a study to investigate whether body mass index (BMI) is associated with menstrual cycle in healthy Chinese women. We would like to warmly invite you to participate this survey and answer all the questions listed below, as many as you can.

All your information is strictly confidential and will be only used for this study. Only research team member is able to access to the data and no other public party will be involved in this study.

We much appreciate your time on completion of this survey. If you have any question, please contact to Dr. Yunhui Tang by 86-13817987805 for assistant. This study was approved by the ethics boards of The Hospital of Obstetrics & Gynaecology of Fudan University and Tengzhou Central People’s Hospital.

**Questions**

1. Your age
2. What are your current height and weight?
3. How many did pregnancy you have?
4. How many live births did you have?
5. The average length of menstrual cycles (days):
6. The average length of menses (days):
7. How many pads do you use during your menses (average)?
8. The amount of blood loss during your menses (please circle one):

Less Medium Heavy

1. Do you have any dysmenorrhea? (please circle one)

Yes: Mild or Severe NO

1. Do you have any chronic gynaecological diseases or other endocrinological disorders, such as uterine fibroids, endometriosis, PCOS, medication for menstruation, irregular menstruation? (please circle one)

Yes NO

Thanks again for your time.

Research Team: Drs. Yunhui Tang, Yan Chen, Qi Chen
